# Outcomes of One‐Piece and Two‐Piece Dental Implants After 15–17 Years: Follow‐Up of a Randomized Clinical Trial

**DOI:** 10.1111/cid.70157

**Published:** 2026-05-13

**Authors:** Miha Pirc, Kevser Pala, Fabiene Rusch, Ronald E. Jung, Daniel S. Thoma, Marc Balmer

**Affiliations:** ^1^ Clinic of Reconstructive Dentistry, Center for Dental Medicine, University of Zurich Zurich Switzerland; ^2^ Faculty of Medicine, University of Ljubljana Ljubljana Slovenia; ^3^ Department of Restorative Dentistry and Biomaterials Sciences Harvard School of Dental Medicine, Harvard University Boston Massachusetts USA; ^4^ Department of Periodontology Research Institute for Periodontal Regeneration, College of Dentistry, Yonsei University Seoul Korea

## Abstract

**Objectives:**

To compare the biological, technical, and esthetic outcomes of one‐piece and two‐piece dental implant systems after 15–17 years in the follow‐up of a previously conducted randomized clinical trial.

**Material and Methods:**

This study represents the 15–17‐year follow‐up of a previously conducted randomized clinical trial included 60 patients requiring dental implant therapy. They were randomly assigned to receive either a one‐piece (STM) or two‐piece (BRA) implant system. Final restoration delivery served as baseline, after which all patients entered a structured maintenance program. Clinical re‐evaluations were performed at 1, 5, 10, 12, 15, and 17 years. Biological, technical, radiographic, and esthetic parameters were assessed at each follow‐up visit using standardized clinical and radiographic protocols.

**Results:**

Thirty‐nine patients with 95 implants, all of whom had completed a 15‐ or 17‐year follow‐up visit, were included in the final analysis (STM: 22 patients/45 implants; BRA: 17 patients/50 implants), with a mean follow‐up of 16.4 ± 1.1 years. The cumulative implant survival rate was 95.0% (STM: 91.8%; BRA: 98.0%). Marginal bone levels at 15–17 years were 0.08 ± 1.15 mm (STM) and 1.53 ± 0.81 mm (BRA). Clinical parameters remained stable in both groups, although BOP was higher in the STM group. Peri‐implant mucositis was observed in 49.7% of all implants and peri‐implantitis in 13.3%, with higher rates in the STM group. Technical complications occurred in 28 implants (STM: 23; BRA: 5), with the STM group showing a higher complication rate (implant level: 35.4% vs. 5.8%). Esthetic observations included visible crown margins in 62 implants and mucosal shimmering in 54 implants, with no implant fractures reported.

**Conclusions:**

Both implant systems demonstrated favorable long‐term outcomes with high survival rates. While the one‐piece system showed superior marginal bone preservation, it was associated with a higher rate of technical complications.

**Trial Registration:** ClinicalTrials.gov identifier: KEK‐ZH‐Nr 2014‐0201

## 1 Introduction

Implant therapy has become an essential treatment modality in reconstructive dentistry, offering highly predictable and effective solutions for a wide range of clinical indications. Treatment options today extend from single‐tooth replacements to comprehensive rehabilitation of partially and fully edentulous patients with fixed or removable prostheses. With advancements in implant design and materials, implant dentistry has evolved significantly, enabling clinicians to address increasingly complex cases with greater precision.

As the field of implantology continues to progress, a diverse array of implant systems has emerged, each varying in terms of surface characteristics, macro‐ and microgeometry, materials, and implant‐abutment connection types. This increasing variety presents clinicians with complex decision‐making processes when selecting an appropriate solution for individual patients. Beyond surgical and prosthetic considerations, system selection must be guided by robust scientific evidence. Critical factors include long‐term survival and complication rates, biological and technical risks, and the continued availability of implant components over time [1].

One of the most significant design differences between implant systems is the distinction between one‐piece and two‐piece implants. The two‐piece implant system, initially introduced to support submerged healing protocols [2], allows for osseointegration in a protected submucosal environment. This concept was originally based on the assumption that exclusion from the oral environment during the early healing phase would facilitate predictable osseointegration [3]. In contrast, the one‐piece system, introduced shortly thereafter [4], integrates both the implant and abutment into a single structure, enabling transmucosal healing and eliminating the need for a second‐stage surgical procedure. In addition to surgical considerations, fundamental differences between one‐piece and two‐piece implant designs relate to the presence of an implant–abutment interface. In two‐piece implant systems, a microgap at this interface may allow bacterial penetration and may be associated with micro‐movements under functional loading. Experimental investigations have demonstrated that these phenomena may influence the biological response of peri‐implant tissues and contribute to marginal bone remodeling around implants [5]. In contrast, one‐piece implant systems eliminate this interface, which has been suggested as a potential biological advantage with regard to peri‐implant tissue stability.

Long‐term studies have demonstrated high survival rates for both implant types. However, most investigations have focused primarily on surrogate endpoints such as implant survival or marginal bone level changes [6, 7]. Despite the widespread use of both implant systems, comparative studies evaluating their overall performance including biological, technical, and esthetic complications over extended observation periods remain limited [8, 9].

Moreover, it remains unclear to what extent the fundamental design differences between one‐piece and two‐piece implants may influence the long‐term stability of peri‐implant tissues, prosthetic complications, and patient‐centered outcomes. Short‐ and medium‐term studies suggest similar survival outcomes between the two systems, however, data extending beyond 10 years of follow‐up are scarce [1, 6, 7]. In addition, survival alone does not fully encapsulate the functional and esthetic demands placed on implant‐supported restorations. Biological complications, and technical complications, such as prosthetic component fractures or screw loosening, are significant factors influencing the overall success of implant therapy [10, 11].

Given these considerations, there is a critical need for long‐term comparative data evaluating not only survival, but also biological stability, prosthetic performance, and esthetic outcomes associated with one‐piece and two‐piece implant designs.

Therefore, the aim of the present study was to compare biological, technical, and esthetic outcomes of one‐ and two‐piece dental implant systems over an observation period of 15 to 17 years.

## 2 Material and Methods

### 2.1 Study Design

**FIGURE 1:**
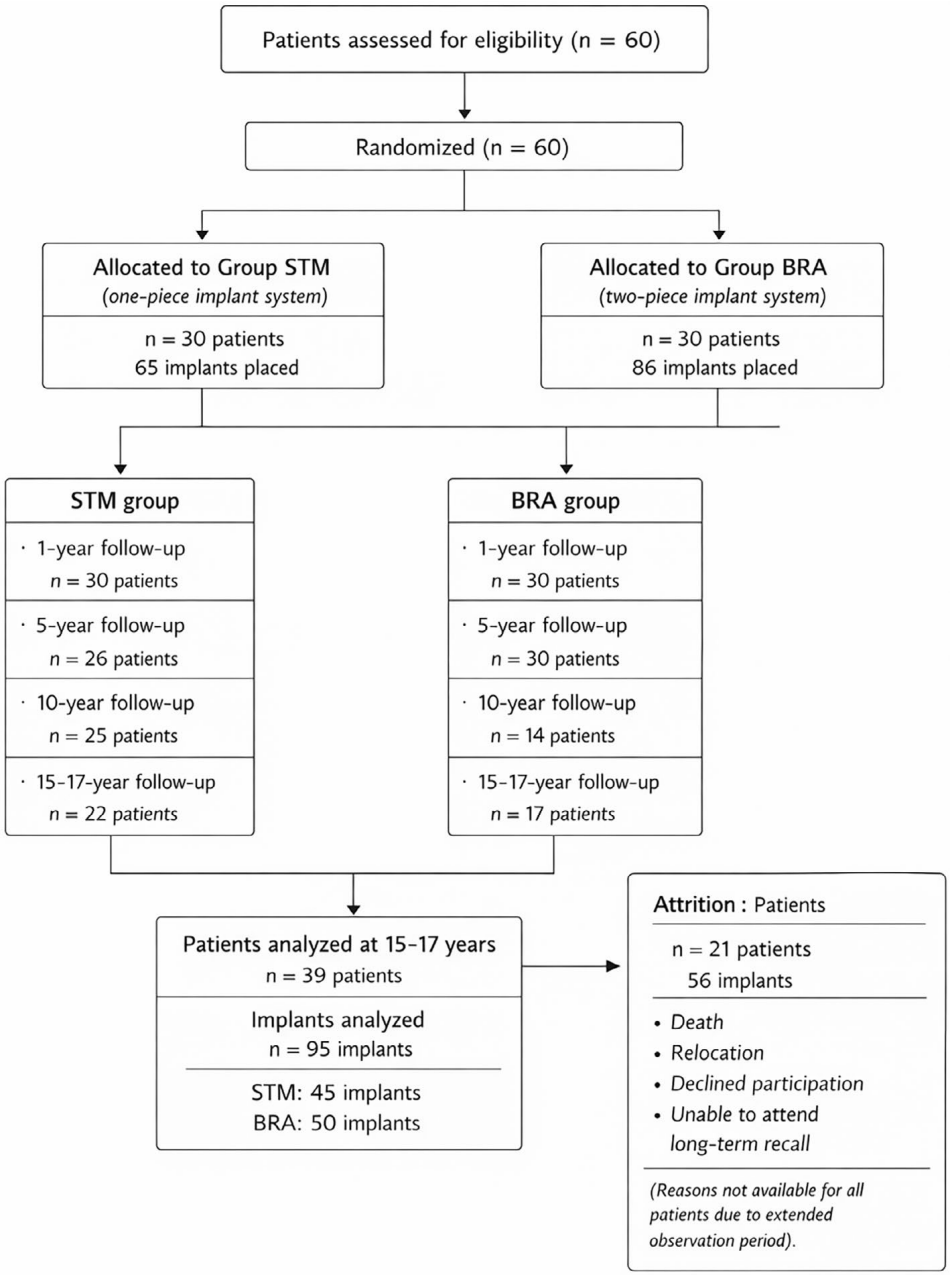
CONSORT flow diagram illustrating patient enrollment, randomization, allocation to Group STM and Group BRA, follow‐up visits, and final analysis over the 17‐year observation period.

This study represents the 15–17‐year follow‐up of a previously conducted randomized clinical trial and received approval from the local ethics committee as a long‐term non‐interventional investigation, given that the reported data represent a continuation of patient follow‐up within the original trial (KEK‐ZH‐Nr 2014‐0201). The specific protocol, inclusion and exclusion criteria were described in detail in the previous publications [12, 13] (Figure 1).

In brief, 60 patients seeking dental implant therapy at the Clinic for Reconstructive Dentistry, Center of Dental Medicine, University of Zürich, Switzerland were included in the study. All patients signed an informed consent and were then randomly allocated to receive either the one‐piece (Institute Straumann, Basel, Switzerland; STM) or two‐piece implant system (Brånemark, Nobel Biocare, Zürich, Switzerland; BRA). At the time of placement, the one‐piece implants were characterized by a sandblasted, large‐grit, acid‐etched (SLA) surface, whereas the Brånemark implants exhibited the TiUnite anodized surface. Both implant systems therefore represented surface‐modified implant designs that were widely used at the time of study initiation. Randomization was performed using a computer‐generated randomization list.

### 2.2 Inclusion and Exclusion Criteria

Inclusion criteria:

- medically healthy
- older than 18 years

Exclusion criteria:

- local or general contraindications for implant therapy
- systemic medical conditions
- drug abuse
- local jaw pathology

No restrictions were made regarding the location of the implants and type of restorations.

### 2.3 Clinical Procedures

All implant surgeries were performed according to the manufacturer guidelines. Implants were placed with the border between the rough and smooth surface at the bone crest (STM group) and with the shoulder of the implant at the bone crest (BRA group). When required for prosthetic considerations, implants were allowed to be placed in a slightly subcrestal position. In case of insufficient bony volume, bone‐grafting procedures were performed either prior to or simultaneously with implant placement. Subsequently, implants were left for transmucosal or submerged healing, depending on the dental surgeon's decision.

### 2.4 Follow‐Up Examinations and Outcome Measures

The delivery of the final restoration served as baseline. All patients were placed in an individually designed maintenance program with regular visits to the dental hygienists. Patients were recalled at 1, 5, 10, 12, 15 and 17 years after the delivery of the final restoration.

The following biological, technical, and esthetic parameters were assessed at baseline and all follow‐up appointments. Due to the inherent structural differences between the one‐piece and two‐piece implant systems, blinding of the clinical examiners during follow‐up examinations was not feasible. The implant type could be identified during the clinical evaluation based on the implant‐abutment configuration and the prosthetic reconstruction.

#### 2.4.1 Biological Outcome Measures

The clinical parameters measured at six sites with a UNC‐15 periodontal probe (Hu‐Friedy, Chicago, IL, USA) were:

- Probing pocket depth (PPD)
- Clinical attachment level (CAL)
- Plaque control record (PCR) [14]
- Bleeding on probing (BOP) [15]

In addition, the incidence of biological complications was assessed at the follow‐up visits or in case patients came in for an extra visit.

– Peri‐implant mucositis was defined as clinical signs of inflammation without crestal bone loss and bleeding on probing > 50% of the sites at a given implant.
– peri‐implantitis was defined as bleeding on probing > 50% in conjunction with crestal bone loss ≥ 2 mm and at the follow‐up appointments [16, 17].

#### 2.4.2 Radiographic Assessment

Intraoral radiographs of all implants were taken at the baseline and all the follow‐up appointments using a paralleling technique with Rinn‐holders. All x‐rays were then digitalized and imported to open‐source software (Image J, National Institutes of Health, Bethesda, Maryland, USA), with which marginal bone level changes were calculated. The known distance between the threads of the implants served as a reference for image calibration and the determination of the exact magnification of the images. Marginal bone levels were measured relative to the implant reference point (shoulder for BRA, rough–smooth border for STM). Bone level changes were then calculated as differences from baseline to each follow‐up.

#### 2.4.3 Technical Complications

All technical complications such as implant fracture, fracture or loosening of the prosthetic screw or abutment, framework fractures, chippings, and loss of retentions were continuously documented in the patient records and assessed at the follow‐up appointments.

#### 2.4.4 Esthetic Outcome Measures

Additionally, the following esthetic parameters were assessed:

- visibility of the crown margin
- shimmering of the implant through the mucosa
- level of the contralateral tooth or implant site

### 2.5 Statistical Analysis

The collected data were analyzed using descriptive and inferential statistical methods. Continuous variables are reported as mean values and standard deviations (SD), while categorical variables are presented as frequencies and percentages. Comparisons of baseline characteristics between patients completing the 15–17‐year follow‐up and those lost to follow‐up were performed using independent samples *t*‐tests for continuous variables and Chi‐square tests for categorical variables.

Due to the exploratory nature of this long‐term follow‐up and the limited number of patients available at the final observation time point, most clinical, radiographic, and technical outcomes were analyzed descriptively. Missing data resulting from patients lost to follow‐up were not imputed; therefore, analyses were based on the available data at each time point.

## 3 Results

### 3.1 Demographic Data

Initially, 60 patients with 151 dental implants (STM: 65, BRA: 86) were included in the study. Six removable (17 implants) and 93 fixed restorations were placed, of which 103 implants had screw‐retained and 31 implants had cement‐retained restorations. A detailed overview of the prosthetic restorations was reported in a previous publication [13]. Details about the implant length, diameter, and distribution were reported in previous publications [12, 13]. Implant lengths were within the range of 6–15 mm, and implant diameters were within the range of 3.3–5 mm. A total of 39 patients with 95 implants were included in the 15–17 year follow up (STM: 22 patients with 45 implants; BRA: 17 patients with 50 implants). The mean follow‐up time was 16.43 years (±1.06), and the mean age of the patients was 70.80 years (±13.49 years). Twenty‐one patients with a total of 56 implants (including the lost implants) did not attend the 15–17 year follow‐up visit.

To assess potential attrition bias, baseline characteristics of patients completing the 15–17‐year follow‐up were compared with those lost to follow‐up (Table S1). No relevant differences were observed with respect to baseline age, sex distribution, implant system allocation, or number of implants per patient, suggesting that the analyzed cohort remained broadly representative of the originally randomized study population. No statistically significant differences were observed between the two groups with respect to baseline demographic characteristics or implant distribution.

### 3.2 Survival Rates

A total of 5 implants were lost during the reported observation period (STM: 4 implants in 3 patients; BRA: 1 implant), resulting in a cumulative implant survival rate of 95%. Divided per group, the survival rate was 91.84% for STM and 98.04% for BRA. The reason for implant loss was peri‐implant disease with progressive peri‐implant bone loss. Due to the limited number of implant losses observed during the study period, implant survival was analyzed descriptively and no formal statistical comparison between the groups was performed.

### 3.3 Radiographic Outcomes

Implant‐level mean crestal bone levels (relative to the reference point) are summarized in Table 1 (note: positive values indicate marginal bone loss). The implant‐level mean marginal bone levels at the FU‐10 were 0.03 mm (±1.08) for STM and 1.38 mm (±0.52) for BRA. The respective values for the 15–17 year observation period were 0.08 mm (±1.15) for STM and 1.53 mm (±0.81) for BRA. The crestal bone level changes on patient level over time are presented in Table 2. Some patients needed to be excluded from the radiographic analysis if no parallel radiograph could be obtained during the study follow‐up visit.

**TABLE 1:** Implant‐level analysis of marginal bone levels (relative to reference point) at baseline and at follow‐ups (5, 10, 15/17 years).

	STM (nBL = 65; n5y = 57; n10y = 53; n15/17y = 31)		BRA (nBL = 86; n5y = 85; n10y = 36; n15/17y = 38)	
	Mean	SD	Mean	SD
Baseline	0.61 mm	0.82 mm	1.07 mm	0.51 mm
FU‐5	0.62 mm	0.77 mm	1.39 mm	0.44 mm
FU‐10	0.03 mm	1.08 mm	1.38 mm	0.52 mm
FU‐15/17	0.08 mm	1.15 mm	1.53 mm	0.81 mm

*Note:* Positive values indicate marginal bone loss relative to baseline.

**TABLE 2:** Patient‐level analysis of mean changes in marginal bone level over time (differences between follow‐up intervals).

	STM (nBL = 30; n5y = 26; n10y = 25; n15/17y = 18)	BRA (nBL = 30; n5y = 30; n10y = 14; n15/17y = 14)
Baseline—FU‐5	0.14 mm	0.32 mm
FU‐5—FU‐10	−0.68 mm	0.03 mm
FU‐10—FU‐15/17	−0.08 mm	0.14 mm

*Note:* Positive values indicate marginal bone loss relative to baseline.

Descriptively, marginal bone levels remained stable in both groups over time. Due to the limited sample size at the final follow‐up visit, no formal inferential comparisons between the groups were performed for these outcomes.

### 3.4 Biological Outcomes

Except for BOP, which was higher in the STM group, the clinical parameters measured (PPD, PCR) were similar for both groups and at all time‐points (Table 3). Reporting on all patients initially included in the study, 75 implants in 34 patients were diagnosed with peri‐implant mucositis (STM: 46 implants in 22 patients; BRA: 29 implants in 12 patients). This resulted in the prevalence of peri‐implant mucositis in 49.67% of all initially included implants (STM: 70.77%; BRA: 33.72%). The patient‐level analysis revealed a peri‐implant mucositis rate of 73.33% (22/30) for the STM group and 40% (12/30) in the BRA group. However, a resolution of the peri‐implant mucositis was observed in 39 of the affected implants and in 21 patients during the observation period, resulting in a remaining peri‐implant mucositis rate of 25.83% of the included implants. The remaining peri‐implant mucositis rates on patient‐level were 2.1% (7/30) in the STM group based on all patients, and 31.82% (7/22) based on the patients that experienced peri‐implant mucositis. The respective patient‐level rates for peri‐implant mucositis cases in group BRA that could not be resolved were 20% (6/30) based on all patients in this group, and 50% (6/12) based on the patients that showed signs of peri‐implant mucositis in this group. During the 15–17 years follow‐up period, 20 implants in 12 patients were diagnosed with peri‐implantitis (STM: 12 implants in 7 patients; BRA: 8 implants in 5 patients). Peri‐implantitis was reported in 13.25% of the initially included implants. The implants diagnosed with peri‐implant disease were treated following a standard protocol with non‐surgical and/or surgical therapy. The patient‐level analysis revealed that 7 out of the originally included 30 patients in the STM group had peri‐implantitis, resulting in a patient‐level peri‐implantitis rate of 23.33%. In the BRA group, 5 patients out of the initially included 30 experienced peri‐implantitis, which equaled a patient‐level peri‐implantitis rate of 16.67%.

**TABLE 3:** Implant‐level analysis of biological complications.

		STM (nBL = 65; n5y = 57; n10y = 53; n15/17y = 31)		BRA (nBL = 86; n5y = 85; n10y = 36; n15/17y = 38)	
		Mean	SD	Mean	SD
PPD	Baseline	3.04 mm	0.67 mm	2.98 mm	0.65 mm
	FU‐5	3.45 mm	0.80 mm	3.36 mm	0.93 mm
	FU‐10	3.75 mm	1.20 mm	3.29 mm	0.95 mm
	FU‐15/17	3.79 mm	1.01 mm	3.51 mm	1.06 mm
PCR	Baseline	14.73%	26.33%	11.91%	19.94%
	FU‐5	20.97%	30.38%	20.93%	28.33%
	FU‐10	22.66%	24.80%	18.05%	26.46%
	FU‐15/17	14.34%	24.82%	13.18%	19.77%
BOP	Baseline	30.67%	26.92%	28.89%	22.40%
	FU‐5	41.53%	34.02%	31.10%	22.54%
	FU‐10	54.62%	20.82%	49.84%	18.94%
	FU‐15/17	45.63%	30.81%	38.33%	25.37%

*Note:* Probing pocket depth (PPD), plaque control record (PCR), bleeding on probing (BOP) at baseline and at 5‐, 10‐ and 15/17‐year follow up visits reported as means and standard deviations (SD).

The clinical parameters assessed showed comparable trends between the two implant systems over time. No statistically significant differences were observed between groups for the reported clinical parameters.

### 3.5 Technical Outcomes

During the 15–17 years observation period, a total of 28 implants in 13 patients showed 40 technical complications (STM: 34 on 23 implants/9 patients, BRA: 6 on 5 implants/4 patients). This analysis presents an overview of the entire observation period and included also technical complications in implants/patients that dropped out before achieving the 15–17 year follow‐up. From all recorded technical complications, 85% occurred in the STM group (34/40). On the patient level, 9 out of 30 patients in the STM group experienced technical complications, resulting in a patient‐level technical complication rate of 30%. With 23 out of 65 implants showing technical complications, the STM group had an implant‐level technical complication rate of 35.38%. The recorded technical complications in STM consisted of 11 matrix renewals (7 implants / 3 patients), 1 ball attachment renewal, 10 screw loosenings (9 implants / 4 patients), 5 minor chippings (4 implants / 2 patients), 2 major chippings (2 implants / 1 patient), 4 losses of screw access whole restorations (4 implants / 2 patients), and 1 debonding. From all observed technical complications, 15% occurred in the BRA group. On the patient level, 4 out of 30 patients in the BRA group experienced technical complications, resulting in a patient‐level technical complication rate of 13.33%. With 5 out of 86 implants showing technical complications, the implant‐level technical complication rate was 5.81% for the BRA group. The following technical complications were observed in the BRA group: 4 minor chippings (3 implants / 2 patients), 1 major chipping, and 1 loss of a screw access whole restoration. During this observation period, no implant fractures, prosthetic screw fractures, abutment fractures, or framework fractures were observed. As already reported in a previous publication (Gamper et al.), a total of two restorations in two patients from STM had to be renewed; one due to a major chipping and one due to an abutment tooth loss of an implant‐tooth‐supported restoration.

Owing to the relatively low frequency of technical complications and the limited number of patients available at the final follow‐up, the analysis of complication rates was performed descriptively.

### 3.6 Esthetic and Further Outcome Measures

During the reported observation period, 54 implants showed a shimmering through the mucosa (STM: 22, BRA: 32). In 62 implants, the crown margin was visible (STM: 31, BRA: 32). The peri‐implant mucosal margin was located more apical compared to the contralateral tooth site at a total of 92 implants (STM: 42, BRA: 50) throughout the observation period.

## 4 Discussion

This study represents the 15–17‐year follow‐up of a previously conducted randomized clinical trial and aimed to evaluate the performance of two implant systems, one‐piece (STM) and two‐piece (BRA), over an extended observation period. The findings predominantly revealed: (i) survival rates of 95%; (ii) stable marginal bone levels in STM and moderate bone level changes in BRA; (iii) slightly higher biological complications in the group STM; (iv) higher technical complications in group STM when compared to the group BRA.

The cumulative survival rate observed in this study was 95%, consistent with previous long‐term reports on implant success rates. Specifically, the survival rates for STM (91.84%) and BRA (98.04%) align with the findings of other long‐term studies on similar implant systems [1, 18, 19, 20]. However, direct comparisons remain limited due to the scarcity of studies evaluating implant survival beyond 15 years.

Marginal bone levels have traditionally been one of the primary criteria for assessing implant success [21]. The current study found minimal bone loss in STM implants (0.08 mm±1.15 mm) and moderate bone loss in BRA implants (1.53 mm±0.81 mm) over the 15–17 year period. Traditionally, the “Brånemark Rule” predicted a progressive but expected bone loss around two‐piece implants, particularly in the first year [22]. The findings in this study are in line with the criteria described at that time for this specific implant design, demonstrating significantly greater stability in the STM group compared to the BRA group. Further factors, such as implant design and surface modalities may have impacted these findings. These results are in agreement with the current literature emphasizing the impact of implant design and implant‐abutment interface on long‐term marginal bone preservation [23]. A potential explanation for the more stable marginal bone levels observed in the one‐piece implant system may be the absence of an implant–abutment interface at the crestal bone level. In two‐piece implant systems, the presence of an implant–abutment junction has been associated with microbial colonization and micro‐movements that may contribute to inflammatory reactions and subsequent bone remodeling. In contrast, the one‐piece design eliminates this interface, which may partly explain the stable marginal bone levels observed during the long‐term observation period. Furthermore, the design of one‐piece implants aims to position the implant‐abutment interface further from the crestal bone, which is thought to help preserve the crestal bone level over time by minimizing micro‐movements and stresses at the bone‐implant interface, potentially enhancing long‐term bone stability [2]. Notably, previous reports on the same pool of patients suggest that the soft tissues might to a certain extent compensate for the reduced marginal bone levels, resulting in lower levels of recessions as would be expected due to the changes in the marginal bone levels. Notably, recent studies have suggested that soft tissues may compensate to some extent for marginal bone loss, reducing recession levels despite bone resorption [24]. This highlights the complex interplay between hard and soft tissues around dental implants. In this context, peri‐implant soft tissue thickness has also been suggested as an important modifying factor influencing crestal bone stability. Previous long‐term studies have indicated that thicker peri‐implant mucosal tissues may contribute to more stable marginal bone levels, potentially by accommodating the establishment of the peri‐implant biologic width. Therefore, variations in peri‐implant soft tissue characteristics may partly explain differences in marginal bone remodeling observed between implant systems [25].

The biological outcomes assessed in this study showed a comparable prevalence of peri‐implant mucositis and peri‐implantitis between both groups. A total of 75 implants exhibited peri‐implant mucositis, while peri‐implantitis affected 20 implants. The findings also showed that in a significant percentage of the reported cases, peri‐implant mucositis could be resolved. These results translate to an overall prevalence of peri‐implantitis of approximately 13%, which aligns with existing long‐term data [26, 27, 28]. These findings underscore the importance of stringent maintenance protocols to manage biological complications and prevent implant failure. Furthermore, the ongoing need for treatment in affected cases adds to the economic burden of implant therapy, highlighting the additional costs and appointments associated with maintenance care [10].

Technical complications were documented in 28 implants across 13 patients, with a total of 40 reported events. A higher frequency of complications was observed in STM implants (34 complications on 23 implants), compared to BRA implants (6 complications on 5 implants). The most common technical issues included matrix renewals, screw loosening, and minor chippings. These findings align with previous systematic reviews [29, 30]. The higher rate of technical complications observed in the one‐piece implant system may partly be explained by inherent design characteristics. In one‐piece implants, the prosthetic reconstruction is directly connected to the implant body without the presence of a separate abutment. This configuration may alter load transmission and stress distribution at the restorative interface and may increase the susceptibility of the prosthetic reconstruction to mechanical complications over time. In addition, restorations associated with one‐piece implants are frequently screw‐retained, which may further influence the mechanical behavior of the restorative components during long‐term function. Furthermore, our results confirm prior trials, which have highlighted the potential economic burden of technical complications, reinforcing the need for careful selection of implant systems with long‐term prosthetic stability [7, 9, 31].

From a clinical perspective, the present findings suggest a potential trade‐off between biological and technical outcomes when selecting implant systems. While the one‐piece implant system demonstrated slightly more favorable marginal bone preservation over the long‐term observation period, it was associated with a higher rate of technical complications. Consequently, both biological stability and the potential need for prosthetic maintenance should be considered during treatment planning and implant system selection. It should also be emphasized that the Brånemark implant system evaluated in this study represents an earlier generation of two‐piece implants. Modern implant systems frequently incorporate internal implant–abutment connections and platform‐switching concepts, which may influence both biomechanical behavior and peri‐implant tissue stability. Therefore, the present findings should be interpreted within the context of the specific implant designs investigated and should not be directly extrapolated to modern two‐piece implant systems.

Despite the extended follow‐up period of this randomized clinical trial, several limitations should be acknowledged. The most important limitation relates to the loss to follow‐up over time. A total of 21 patients corresponding to 56 implants did not attend the final 15–17‐year examination, which may introduce attrition bias. Although the overall retention rate remained acceptable considering the long observation period, it cannot be excluded that patients lost to follow‐up may have experienced different clinical outcomes, potentially influencing the reported results and limiting their generalizability.

In addition, the clinical protocol allowed a certain degree of heterogeneity with respect to implant placement depth, the use of bone augmentation procedures, healing protocols, and prosthetic reconstruction types. While such variability reflects routine clinical decision‐making in implant therapy, these factors may have influenced marginal bone levels, biological outcomes, and the occurrence of technical complications. Furthermore, blinding of the clinical examiners was not feasible due to the visible design differences between the implant systems, which may have introduced a potential measurement bias when assessing certain clinical or esthetic parameters. Nevertheless, the variability in treatment approaches likely reflects the heterogeneity encountered in everyday clinical practice and may therefore enhance the external validity of the findings. Finally, the reduced number of patients available for the final analysis limited the statistical power to detect potential differences between the two implant systems, particularly for relatively infrequent events such as peri‐implantitis or specific technical complications. Consequently, the results should be interpreted with caution when comparing complication rates between the groups.

## 5 Conclusions

The results of this study demonstrate that both implant systems evaluated provide reliable long‐term outcomes with high survival rates. The findings suggest that the one‐piece implant system tested may offer advantages in marginal bone preservation. However, this system also exhibited a higher incidence of technical complications. Further research with larger sample sizes and extended follow‐up periods is needed to enhance our understanding of the long‐term performance and reliability of dental implants.

## Author Contributions

**Miha Pirc:** data interpretation, drafting article, critical revision of article, data collection. **Kevser Pala:** data analysis, drafting article, critical revision of article, statistics, data collection. **Fabiene Rusch:** critical revision of article, data collection. **Ronald E. Jung:** concept/design, funding secured. **Daniel S. Thoma:** concept/design, drafting article, critical revision of article, approval of article. **Marc Balmer:** critical revision of article, approval of article.

## Funding

Study was self‐funded by the Clinic of Reconstructive Dentistry, Center for Dental Medicine, University of Zurich, Zurich, Switzerland.

## Consent

All patients signed an informed consent.

## Conflicts of Interest

The authors declare no conflicts of interest.

## Supporting information


**Table S1:** Baseline characteristics of patients completing the 15–17‐year follow‐up versus those lost to follow‐up.

## Data Availability

The data that support the findings of this study are available from the corresponding author upon reasonable request.
