# Supplementary material for: Outcomes of One‐Piece and Two‐Piece Dental Implants After 15–17 Years: Follow‐Up of a Randomized Clinical Trial
Source: Clin Implant Dent Relat Res. 2026 May 13;28:e70157. doi: 10.1111/cid.70157 (PMC13170413; doi:10.1111/cid.70157)
Supplement: Supplementary file 1 — Table S1: Baseline characteristics of patients completing the 15–17‐year follow‐up versus those lost to follow‐up. [file CID-28-0-s001.docx]

*Table S1. Baseline characteristics of patients completing the 15–17-year follow-up versus those lost to follow-up*

| **Variable** | **Completed follow-up** | **Lost to follow-up** | **p-value** |
| --- | --- | --- | --- |
| Patients (n) | 39 | 21 | — |
| Implants (n) | 95 | 56 | — |
| Mean age at baseline (years ± SD) | 50.9 ± 15.1 | 52.8 ± 14.7 | 0.61 |
| Sex (male / female) | 13 / 26 | 7 / 14 | 0.93 |
| Implant system (STM / BRA), n | 22 / 17 | 8 / 13 | 0.41 |
| Implants per patient (mean ± SD) | 2.44 ± 1.52 | 2.67 ± 1.41 | 0.52 |
| Jaw location (maxilla / mandible), implants | 56 / 39 | 31 / 25 | 0.87 |
